# Endophytic Bacteria Induce Thiamine (Vitamin B1) Production in Oil Palm (*Elaeis guineensis*)

**DOI:** 10.21315/tlsr2024.35.1.1

**Published:** 2024-03-30

**Authors:** Nur Asna Faiqah Johari, Aisamuddin Ardi Zainal Abidin, Nur Farhah Nabihan Ismail, Zetty Norhana Balia Yusof

**Affiliations:** 1 Department of Biochemistry, Faculty of Biotechnology and Biomolecular Sciences, Universiti Putra Malaysia, 43400 UPM Serdang, Selangor, Malaysia; 2 Aquatic Animal Health and Therapeutics Laboratory (AquaHealth), Institute of Bioscience, Universiti Putra Malaysia, 43400 UPM Serdang, Selangor, Malaysia; 3 Bioprocessing and Biomanufacturing Research Complex (BBRC), Universiti Putra Malaysia, 43400 UPM Serdang, Selangor, Malaysia

**Keywords:** Endophytes, Gene Expression, Oil Palm, Thiamine, Vitamin B1, Endofit, Ekspresi Gen, Kelapa Sawit, Tiamin, Vitamin B1

## Abstract

Thiamine or vitamin B1 is a micronutrient that has a crucial function in all living organisms and involved in several biochemical reactions. Concerning the capability of thiamine in inducing plant health, a study was carried out by applying bacterial endophytes (*Pseudomonas aeruginosa* and *Burkholderia cepacia* cultures) in four-month-old oil palm seedlings (*Elaeis guineensis*) via soil drenching technique to evaluate the effect towards thiamine. Spear leaves were sampled day 0 to 14 to analyse the expression of gene coding for the first two enzymes thiamine biosynthesis pathway, THI4 and THIC via qPCR analysis. The gene expression by qPCR showed a significant increase of up to 3-fold while high-performance liquid chromatography (HPLC) analysis for quantification of thiamine and its derivatives accumulated ~ 20-fold in total thiamine when compared to control seedlings. However, concentration of thiamine metabolites was negatively correlated with the expression of THIC and THI4 gene transcripts suggesting post-transcriptional regulation mediated by an RNA regulatory element, a thiamine pyrophosphate (TPP) riboswitch. Our findings demonstrated that the application of bacterial endophytes affected thiamine biosynthesis and enhanced overall thiamine content. This might increase the plant’s resistance towards stress and would be useful in oil palm maintenance for maximum yield production.

## Abstract (ms)

Tiamin atau vitamin B1 ialah mikronutrien yang mempunyai fungsi penting dalam semua organisma hidup dan terlibat dalam beberapa tindak balas biokimia. Untuk mengenal pasti keupayaan tiamin dalam mendorong kesihatan tumbuhan, satu kajian telah dijalankan dengan menggunakan kultur bakteria endofit (*Pseudomonas aeruginosa* dan *Burkholderia cepacia*) ke atas anak benih kelapa sawit berumur empat bulan (*Elaeis guineensis*) melalui teknik siraman untuk menilai kesan terhadap penghasilan tiamin. Sampel daun muda telah diambil pada hari 0 hingga 14 untuk menganalisis ekspresi gen yang mengekodkan dua enzim pertama laluan biosintesis tiamin, THI4 dan THIC melalui analisis qPCR. Analisis ekspresi gen oleh qPCR menunjukkan peningkatan ketara sehingga 3 kali ganda manakala analisis HPLC untuk kuantifikasi tiamin dan derivatifnya menunjukkan peningkatan ~ 20 kali ganda dalam jumlah tiamin jika dibandingkan dengan anak benih kawalan. Walau bagaimanapun, jumlah metabolit tiamin dikaitkan secara negatif dengan ekspresi transkrip gen THIC dan THI4 yang mencadangkan kawalan pasca transkripsi yang dimediasi oleh unsur pengawalseliaan RNA, suis-ribo tiamin pirofosfat (TPP). Kajian ini menunjukkan bahawa penggunaan kultur endofit bakteria dapat memberi kesan kepada biosintesis tiamin dan meningkatkan kandungan tiamin secara keseluruhan. Keadaan ini boleh meningkatkan daya tahan tumbuhan terhadap tekanan dan berguna dalam pengawalan pokok kelapa sawit untuk pengeluaran hasil yang maksimum.

**Highlights**

- The expression of gene transcripts coding for the first two enzymes in the thiamine biosynthesis pathway, THI4 and THIC showed significant increase of up to 3-fold.
- Thiamine and its derivatives accumulated up to 20-fold.
- Concentration of thiamine metabolites was negatively correlated with the expression of THIC and THI4 gene transcripts suggesting posttranscriptional regulation mediated by an RNA regulatory element, a thiamine pyrophosphate (TPP) riboswitch.
- The application of bacterial endophytes *Pseudomonas aeruginosa* and *Burkholderia cepacia* affected thiamine biosynthesis and enhanced overall thiamine content.

## INTRODUCTION

**Figure 1:**
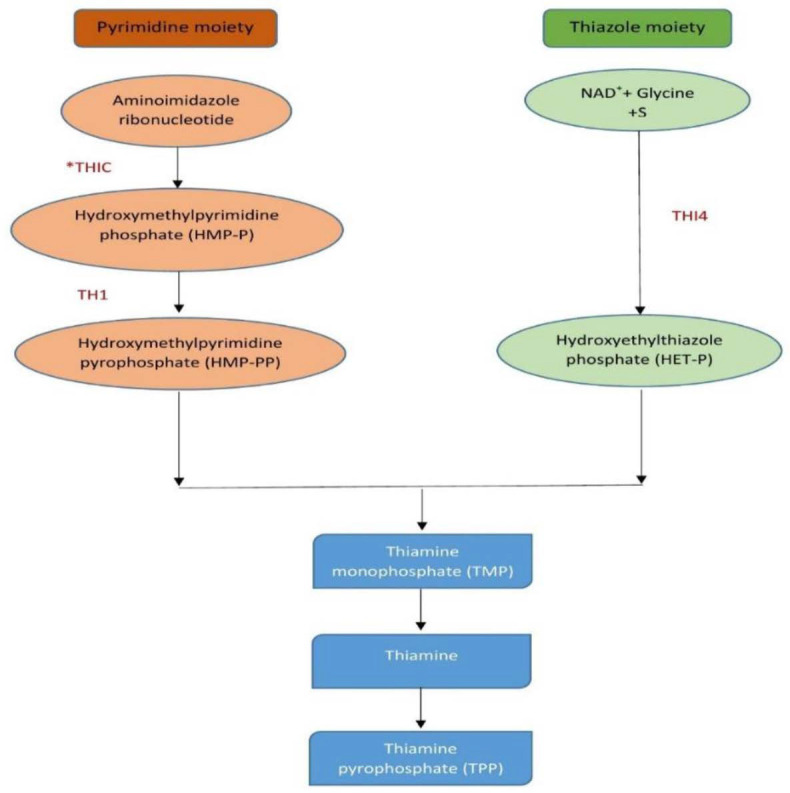
Overview of plant metabolic pathway of thiamine biosynthesis. THIC = Phosphomethylpyrimidine synthase; THI4 = Thiamine thiazole synthase; TH1 = Thiamine bifunctional enzyme.

Mammals, including humans, lack the ability to biosynthesize thiamine and therefore plants are one of the major dietary sources of this micronutrient. Thiamine serves as a cofactor and has a central role in central metabolic pathways such as tricarboxylic acid cycle (TCA) and pentose phosphate pathway (PPP) (Goyer 2010). *De novo* biosynthesis of thiamine in plants utilised the combination of bacterial and yeast pathways involving sub-cellular splitting during the process (Pourcel *et al*. 2013). Thiamine comprises a thiazole ring linked to a pyrimidine ring by a methylene bridge (Dong *et al*. 2015). The formation of thiamine can be achieved *via* condensation of two types of moieties which are hydroxyethylthiazole phosphate (HET-P) and hydroxymethylpyrimidine pyrophosphate (HMP-PP). These moieties will then be coupled to form thiamine monophosphate (TMP). Phosphorylation of thiamine to form thiamine pyrophosphate (TPP) occurs in the cytosol. Therefore, thiamine (or TMP) is exported from the chloroplast to the cytosol for the latter step to be accomplished. THIC or HMP-P synthase is the first enzyme in the pyrimidine branch in the thiamine biosynthesis pathway, while THI4 or HET-P synthase is the first enzyme in the thiazole branch of the pathway (Pourcel *et al*. 2013). Fig. 1 summarises the thiamine biosynthesis pathway in plants.

Many current studies are revealing the role of thiamine in response to abiotic and biotic strssess. Meanwhile, previous studies suggested that boosting thiamine content could increase the plant’s resistance towards stresses (Dong *et al*. 2015). Biotic stress is a condition caused by living organisms such as animals, bacteria, viruses and parasites (Shanker & Venkateswarlu 2011). On the other hand, environmental factors such as drought and flood are a few abiotic stresses which cause morphological, physiological, biochemical, and molecular changes in plants (Shanker & Venkateswarlu 2011). Rapala-Kozik *et al*. (2012) examined the response of genes involved in vitamin B1 biosynthesis under several different abiotic stresses in *Arabidopsis* such as salt and osmotic stress leading to the upregulation of the expression of thiamine biosynthesis genes namely THI4, THIC, TPK and TH1. A study published by Yusof *et al*. (2015) exhibited the effect of *Ganoderma boninense* infection towards the expression of THIC gene in oil palm (*Elaeis guineensis*) which suggested that thiamine may play an important role in adaptation to biotic stress. The application of the endophytic fungus, *Hendersonia toruloidea* has shown to increase both the gene expression of the first two enzymes in the pathway as well as the overall thiamine content in oil palm (Kamarudin et al. 2017a; Kamarudin et al. 2017b). Moreover, the application of abiotic stresses to induce salinity, osmotic and oxidative stresses have also been proven to affect thiamine biosynthesis in oil palm (Yee *et al*. 2016; Abidin *et al*. 2016; Rahman *et al*. 2017; Idris *et al*. 2018). Induction of thiamine production and the expression of genes coding for enzymes in the pathway have also been identified in microalgae and cyanobacteria (Fern *et al*. 2017). A study on Malaysian indigenous microalgae and cyanobacterium showed that abiotic stresses induce total phenolic, total flavonoid, and antioxidant properties in these organisms (Azim *et al*. 2018). These findings have contributed to the understanding of thiamine’s indirect role in enhancing antioxidative capacity in plants, which is important in defence responses (Zhao *et al*. 2014). However, the studies of thiamine in oil palm have yet to be expanded further. Recently, a riboswitch has been discovered in THIC gene of oil palm as has been described in other plants (Subki *et al*. 2020; Subki & Yusof 2018). Besides that, the application of exogenous thiamine to oil palm seedlings has shown to regulate thiamine production in oil palm (Subki *et al*. 2018).

At present, palm oil is the most produced and consumed vegetable oil in the world (Hansen *et al*. 2015). In Malaysia, the palm oil industry has secured substantial income for the national economy (Ludin *et al*. 2014). The productivity of oil palm is strongly influenced by environmental stressors, specifically infections caused by the pathogenic fungi *G. boninense*, that eventually affect crude palm oil (CPO) production (Kamil & Omar 2016).

To address the limiting constraint due to *Ganoderma* infection, various measures have been taken, but the fungal infections have not been eradicated completely. Therefore, researches have been focusing on the detailed molecular mechanism of the plant-pathogenic interaction (Ho & Tan 2015). Since environmental stresses have become the major threat to oil palm crops, the application of beneficial endophytic bacteria in the plant might be a good strategy. This is also due to the fact that it is an environmentally friendly solution to overcome diseases and stresses in the oil palm industry.

Endophytes are a group of microorganisms that colonize the interior part of a plant without causing any harm to the host. A study by Sapak *et al*. (2008) showed that oil palm seedlings exhibited an increased resistance towards basal stem rot disease when applied with bacterial endophytes. This study aimed to examine the responses of oil palm seedlings towards endophytic colonisation, specifically on the expression of the first two enzymes in the thiamine biosynthesis pathway, THI4 and THIC. Total thiamine accumulation was also quantified using HPLC and were compared with untreated oil palm seedlings. Thus, the interrelation between the expression of thiamine biosynthesis genes with the production of thiamine as well as its intermediates would assist in understanding the symbiotic relationship of endophytes and oil palm using thiamine metabolism.

## MATERIALS AND METHODS

### Plant Materials

Four-month-old oil palm seedlings of *Tenera* sp. were obtained from Felda Enstek Nilai, Malaysia. The seedlings were grown under nursery conditions at Faculty of Biotechnology and Biomolecular Sciences, Universiti Putra Malaysia. The seedlings were acclimatised for one week for adaptation under constant conditions and were watered with 125 mL of distilled water constantly at 5:00 p.m. every day.

### Bacterial Strain Growth and Culture Maintenance

Two types of bacteria were used in this study. *Pseudomonas aeruginosa* and *Burkholderia cepacia* were obtained from the Department of Plant Protection, Faculty of Agriculture, Universiti Putra Malaysia, Selangor, Malaysia. The maintenance was done based on the method established by Sapak *et al*. (2008) with some modifications. The cultures of *P. aeruginosa, B. cepacia*, and a mixture of both cultures were grown in nutrient broth (Merck, Germany) for 48 h at room temperature at the speed of 130 rpm. The concentrations of cultures were standardised to 10^8^ CFU/mL. For the mixture treatment, both *P. aeruginosa* and *B. cepacia* were mixed at equal volume.

### Bacterial Application to Oil Palm Seedlings

The establishment of the *P. aeruginosa* and *B. cepacia* in oil palm seedlings was done using a complete randomised design (CRD) with three biological replicates. Approximately 200 mL of *P. aeruginosa, B. cepacia*, and mixed cultures were applied by drenching technique (Sapak *et al*. 2008). Distilled water-treated seedlings were used as control. Time point samplings were done on days 0, 1, 3, 7 and 14.

### RNA Isolation and cDNA Synthesis

Spear leave tissues (0.5 g) were ground in liquid N_2_ and transferred in 2 mL tubes. Extraction buffer (800 μL) was added and vigorously mixed by vortexing. About 500 μL phenol-chloroform mixture (1:1) of pH 4.7 was added. The sample was mixed well and centrifuged at 13,000 × *g* for 15 min at 4°C. Supernatant was transferred into new tubes. Isopropanol (600 μL) and 500 μL of 1.2 M NaCl was then added and mixed by inversion. Incubation was performed on ice for 15 min before being centrifuged at 13,000 × *g* for 15 min at 4°C. Supernatant was discarded and the pellet was washed with 70% ethanol. The pellet was air-dried before being suspended in 25 mL dH_2_O. DNase treatment was carried out according to Novagen DNase I RNase-free Protocol (Novogen, USA). Reverse Transcription was performed using Tetro cDNA Synthesis Kit (Bioline, USA). Specific primers of THI4 and THIC were designed using Primer Premier 6.0 (Primer Biosoft, Palo Alto, USA) (Kamarudin *et al*. 2017a).

### Quantitative Real-Time Polymerase Chain Reaction (qPCR)

Gene expression of THIC and THI4 transcripts was then quantified using CFX Connect™ Real-Time PCR machine (Biorad, UK). Real-time PCR reactions were performed in triplicate using 0.4 μg of cDNA template, 0.4 μM of forward and reverse primers for each respective gene, and 1× SensiFAST^TM^ SYBR No-ROX green PCR master mix (Bioline, USA) in a 20 μL volume. qPCR analysis was only done on oil palm samples treated with *B. cepacia* and *P. aeruginosa*. The expression of the genes of interest (THIC and THI4) were analysed as described by Pfaffl (2004).

### DNA Sequencing

Purified PCR products were sequenced using 1st Base Sequencing Service (1st Base, Singapore). Basic Local Alignment Search Tool (BLAST) (http://blast.ncbi.nlm.nih.gov) was used to recover the gene of interest.

### Quantification of Thiamine Metabolites

B1 vitamers (TMP, TPP and thiamine) were extracted from 2.5 g spear leaves of oil palm samples in 0.1 N hydrochloric acid. The mixture was vortexed and incubated at 37°C overnight. Then, the supernatant was collected. Further derivatisation of the extract was carried out using potassium ferricyanide and sodium hydroxide to oxidise the metabolite to its fluorescent derivative, thiochrome. The calibration curves of targeted metabolites (Thiamine-HCl, TMP and TPP) were plotted through standard preparation at 10 ppm, 2 ppm, 1 ppm, 0.4 ppm and 0.2 ppm. Quantification of thiamine vitamers was performed by integrating the corresponding fluorescent peak area extrapolated from standard curves. The column used during the study was a C18 Kinetex Column (100 × 4.6MM, 5 μm particle diameter) (Kamarudin *et al*. 2017a).

## RESULTS

### Expression of Thiamine Biosynthesis Genes in Oil Palm Seedlings Upon Bacterial Application

**Figure 2:**
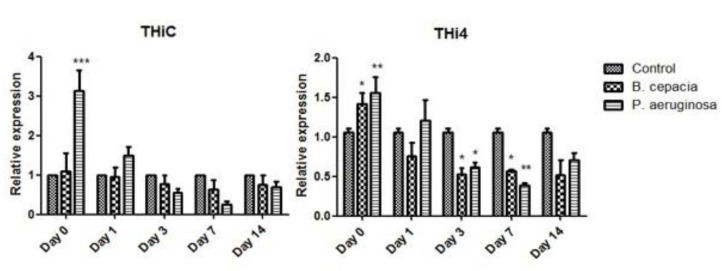
Analysis of THIC and THI4 gene expression under normal conditions, treated with *B. cepacia* and *P. aeruginosa* using qPCR. *N* = 3 replicates. Error bars represent SEM. *P < 0.05, **P < 0.01 and ***P < 0.001 versus control (untreated cultures), using one-way ANOVA (Dunnett post-test) on the raw data.

To quantify and examine the effect of targeted gene expression on the colonisation of endophytes, namely, *B. cepacia* and *P. aeruginosa*, two thiamine biosynthesis genes (THIC and THI4) were analysed using qPCR. The analysis of THIC and THI4 gene expression in oil palm seedlings under normal conditions is presented in Fig. 2. Upon the application of the endophyte *P. aeruginosa*, both THI4 and THIC showed a significant increase in gene expression compared to control (untreated oil palm seedlings) at day 0. However, the expression declined after day 0 until day 7. It is also worth noting that no significant differences in expression could be seen in THIC gene expression. Meanwhile, the application of endophyte *B. cepacia* showed a significant decrease in THI4 gene expression starting from day 0 to day 7. Notably, THI4 gene transcript was found to be higher than THIC in both endophytes’ applications. These results agree well with existing studies on *Chlamydomonas* (Moulin *et al*. 2013) and cassava (Mangel *et al*. 2017). This may be due to the involvement of the endophyte in stimulating or repressing the thiamine biosynthesis machinery in host plants.

### Quantification of Vitamin B1 in Oil Palm Seedlings Upon Bacterial Application

**Figure 3:**
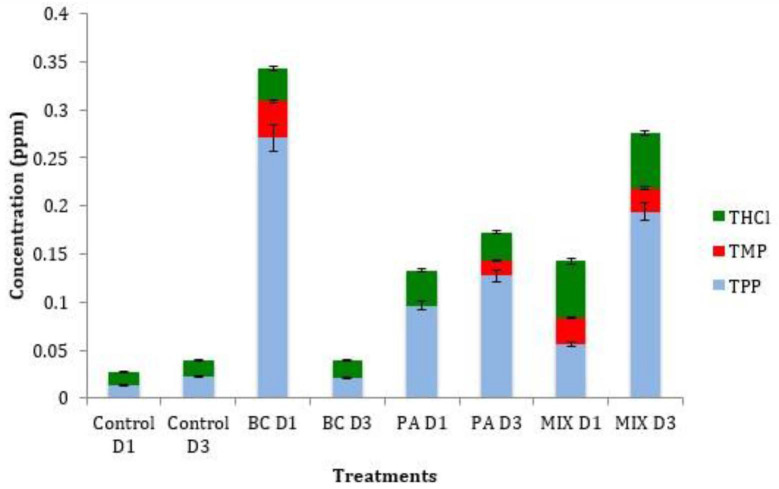
Accumulation of thiamine vitamers (Thiamine, TMP and TPP) in oil palm seedlings upon bacterial induction (*P. aeruginosa*, *B. cepacia* and mix). Data presented are the mean SD of three biological replicates. Significant differences (*P* < 0.05; Tukey’s multiple comparison test). D1 = Day 1, D3 = Day 3, BC = *B. cepacia*, PA = *P. aeruginosa*.

In the present study, the quantification of thiamine and its metabolites (TMP and TPP) was evaluated to understand the relationship between gene expression and the downstream regulation of the thiamine biosynthesis pathway. Both single treatments of *B. cepacia* and *P. aeruginosa* have exhibited an increase in total thiamine content at day 1 post-treatment as shown in Fig. 3. It is also interesting to note that the single treatment of *B. cepacia* was superior to the mixed treatment as it exhibited higher thiamine content (~20-fold) compared to the mixed treatment with ~ 8.6-fold increase. This was also observed in a study performed by Bandara *et al*. (2006), in which plant inoculation with mono-cultures produced more indoleacetic acid compared to mixed cultures. Accumulation of thiamine is beneficial to the plant as it will increase the plant’s metabolic fitness through the production of reactive oxidative signalling (Rapala-Kozik *et al*. 2011).

## DISCUSSION

Declination of the expressions is possibly a result of the adaptation or regulation effect of thiamine biosynthesis genes as suggested in previous studies (Rapala-Kozik *et al*. 2012; Abidin *et al*. 2016). This is consistent with what has been found in previous studies on oil palm seedlings subjected to *G. boninense* infection and abiotic stresses (Yusof *et al*. 2015; Abidin *et al*. 2016). As the thiamine biosynthesis pathway is initiated from two branches, *B. cepacia* may perturb THIC gene expression by providing the source HMP-P or HMP-PP to the oil palm seedlings, which has led to no significant increase of THIC gene expression. Throughout the period, both genes were downregulated under the application of *B. cepacia* despite the initial increase of THIC gene expression.

The highest thiamine accumulation can be seen in the mixed treatment at day 1 with up to 4.3-fold increment. However, the relative accumulated thiamine is lower compared to TPP. This discovery is in line with a study by Mangel *et al*. (2017) that suggests the curb might be due to an insufficient supply of hydroxyethylthiazole phosphate (HET-P) to form thiamine. THI4 gene (responsible for HET-P production (Fig. 1) expression was significantly increased under both applications of endophytes on day 0 and day 1 (Fig. 2). Vitamin B1 is mainly present as a phosphorylated ester and primarily as TPP in microalgae, *Arabidopsis* and rice (Rapala-Kozik *et al*. 2012; Moulin *et al*. 2013; Pourcel *et al*. 2013; Dong *et al*. 2016). The thiamine conversion process to its active form in order to be transported or act as a coenzyme might be an explanation of the lower thiamine accumulation compared to TPP. Therefore, TPP accumulation in the spear leaves of oil palm seedlings might be due to the need for it to be transported to the root where the colonisation of the endophytes is taking place. A similar pattern of quantification of vitamin B1 was also observed in cassava leaves where the TPP accumulation was high (Mangel *et al*. 2017). The increment of thiamine level in the plant when subjected to abiotic stress strongly correlated with the overexpression of the upstream genes (Bocobza *et al*. 2013).

The low or barely detectable content of TMP metabolites in oil palm suggests that regulation of thiamine metabolism was controlled by TPP riboswitch feedback mechanism. When there is a significant accumulation of TPP in the cellular environment, the TPP metabolite will directly bind to the riboswitch sequence located in the 3′ untranslated (3′-UTR) region of THIC gene causing it to shut down its machinery pathway thus explaining the inclination of TMP accumulation. Another study conducted by Bocobza *et al*. (2007) reported that TPP causes under expression of THIC gene in *Arabidopsis* sp. suggesting the presence of regulatory elements called riboswitch. Feedback regulation conducted by TPP riboswitch was seen to be important to prevent thiamine pool accumulation in the cellular environment (Bocobza *et al*. 2007). Overall, TPP was detectable at a higher level in contrast to TMP and thiamine. The expression of THI4 and THIC gene transcripts were found to be negatively correlated with total thiamine content. This could be explained through the involvement of post-transcriptional regulation mediated by TPP riboswitch (Mangel *et al*. 2017).

## CONCLUSION

The present study has demonstrated a differential response in gene expression in terms of single inoculation and mixed inoculation of *B. cepacia* and *P. aeruginosa*. Overall, it can be concluded that application of bacterial endophytes enhanced thiamine biosynthesis and subsequently thiamine content in oil palm seedlings. Enhancement of thiamine biosynthesis could be an attractive solution to produce stress and disease-tolerant oil palm for maximum yield. Further molecular, biochemical and physiological studies need to be performed to understand the role of thiamine in stress protection in plants, especially useful crops like oil palm.
